# Acute Hepatitis A Causing Severe Hemolysis and Renal Failure in Undiagnosed Glucose-6-Phosphate Dehydrogenase Deficient Patient: A Case Report and Review of the Literature

**DOI:** 10.1155/2021/5512883

**Published:** 2021-06-03

**Authors:** Iman Abutineh, Kyle Kreitman, Jiten P. Kothadia, Bilal Ali, Richa Jain, Ian Clark, Benedict J. Maliakkal, Satheesh P. Nair

**Affiliations:** ^1^Department of Internal Medicine, University of Tennessee Health Science Center, Memphis, TN, USA; ^2^Methodist University Hospital, James D. Eason Transplant Institute, University of Tennessee Health Science Center, Memphis, TN, USA; ^3^Department of Pathology, Memphis Pathology Group, University of Tennessee Health Science Center, Methodist University Hospital, Memphis, TN, USA

## Abstract

Infection with hepatitis A virus is usually a self-limited illness that rarely results in fulminant liver failure. Severe hemolysis is an uncommon complication but has been reported in patients with glucose-6-phosphate dehydrogenase (G6PD) deficiency. Here, we report a case with undiagnosed G6PD deficiency who presented with hyperbilirubinemia, severe hemolysis, and acute renal failure precipitated by acute hepatitis A infection.

## 1. Introduction

Hepatitis A is one of the most frequent causes of foodborne infection, with an estimated 1.5 million cases each year [1]. Infection most commonly manifests with symptoms such as fever, malaise, anorexia, abdominal discomfort, diarrhea, dark-colored urine, and jaundice. Acute hepatitis A is usually a self-resolving illness without chronic sequelae. Acute liver failure develops in approximately one percent of cases [2].

A mild to moderate degree of hemolysis is a known complication of acute viral hepatitis. In a few cases, patients may present with severe hemolysis, which is more frequent in patients with coexisting glucose-6-phosphate dehydrogenase (G6PD) deficiency [2–9]. It has been postulated that viral infection itself can cause severe hemolysis in a patient with G6PD deficiency. In addition, the administration of certain drugs and particular foods may aggravate the condition [2]. It has also been noted that G6PD deficiency impedes the repair of the damaged hepatocytes and can lead to fulminant hepatitis and liver failure in patients with acute viral hepatitis [10, 11]. Here, we describe a patient with undiagnosed G6PD deficiency who presented with hyperbilirubinemia, severe hemolysis, and acute renal failure precipitated by acute hepatitis A infection.

## 2. Case Report

A 55-year-old African American male with a medical history of hypertension, hyperlipidemia, chronic hepatitis B, and well-controlled HIV on ART presented to the hospital with a one-week history of fatigue, fever, myalgias, shortness of breath, and dark-colored urine. He denied any history of alcohol, illicit drug use, or family history of liver disease.

On physical exam, he had scleral icterus and mild lower abdominal pain without guarding, rebound, or rigidity. He was alert and oriented without focal deficit or asterixis. He had no ascites. Vital signs were within normal limits. Lab work was significant for creatinine at 6.38 mg/dL (0.7–1.3), BUN 48 mg/dL (7–18), total bilirubin 51.8 mg/dL (0.2–1), direct bilirubin 40.4 mg/dL (≤0.2), lactate dehydrogenase 2678 unit/L (87–241), haptoglobin <7.7 mg/dL, alkaline phosphatase 195 unit/L, AST 216 unit/L (15–37), ALT 6,244 unit/L (16–61), lactic acid 5.5 mmol/L (0.4–2), WBC 16.6 thou/mcL (4.2–10.2), hemoglobin 7.5 g/dL (12.8–16.4), platelet 344 × 10^3^/microL (150–400), and INR 1.6 (0.8–1). His model for end-stage liver disease (MELD) score was 40. Urine drug screen, acetaminophen, and alcohol level were negative. Serologic studies for *Treponema pallidum*, cytomegalovirus, Epstein–Barr virus, herpes simplex virus, and parvovirus were negative. Influenza and COVID-19 PCR were negative. The hepatitis panel was positive for hepatitis A IgM, hepatitis A total antibody, and hepatitis B core IgG antibody. Hepatitis C antibody, hepatitis E IgG/IgM, and HBV DNA were negative. His autoimmune markers including antinuclear antibody, antismooth muscle antibody, antimitochondrial antibody, and myeloperoxidase autoantibody were negative. Serum ceruloplasmin was also negative. Erythrocyte G6 PD level was normal due to acute hemolysis. A computed tomography (CT) scan of the abdomen and pelvis showed normal appearing liver with nondilated bile ducts and patent hepatic vasculature. A transthoracic echocardiogram (TTE) showed LVEF 60–65% with normal wall motion, no diastolic dysfunction, valvular disease, or thrombus.

**Figure 1:**
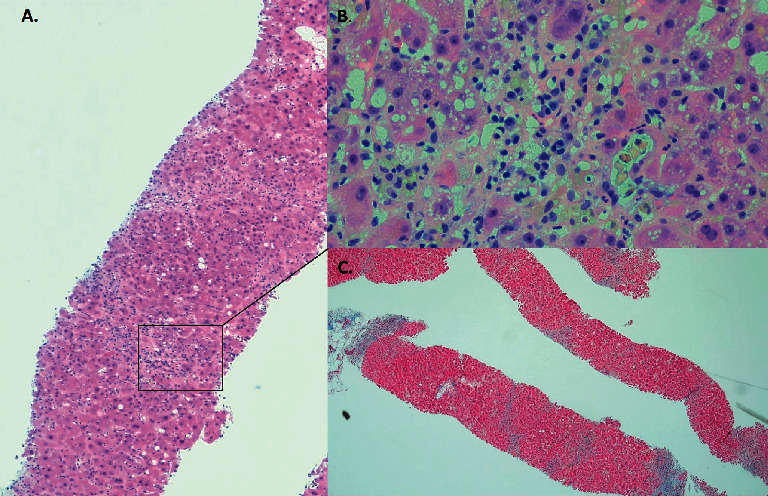
(a) Liver biopsy (H&E, x40) showing bridging necrosis and collapse of parenchyma with cholestasis and sparse inflammatory infiltrate rich in lymphocytes and plasma cells ((b) inset x200). (c) A trichrome stain (x20) showing lack of fibrosis supporting acute nature of the illness.

**Figure 2:**
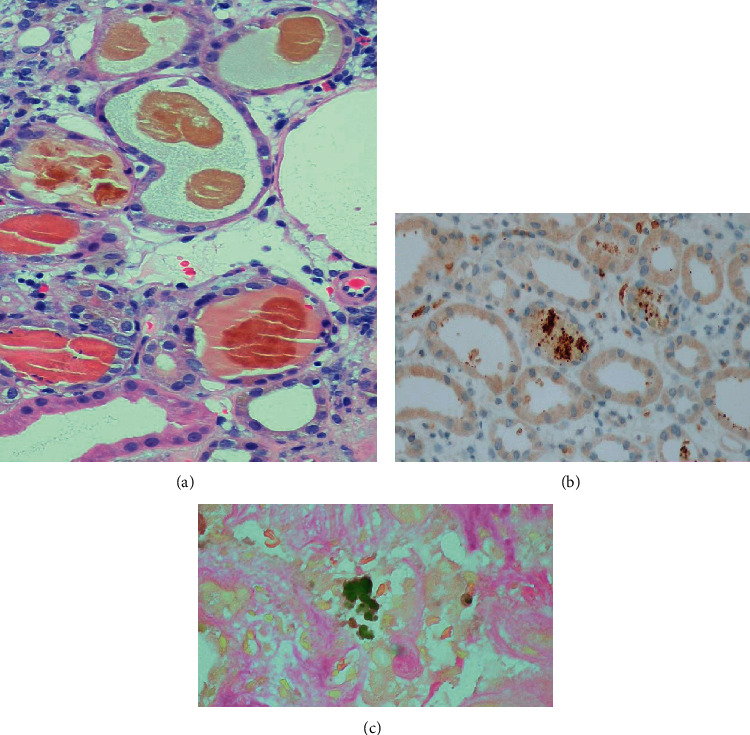
(a) Renal biopsy showing tubular casts comprising of hemoglobin (hemoglobin immunostained (b)) and bile pigment (Fouchet stained (c)). Glomeruli and vasculature were within normal limits.

**Figure 3:**
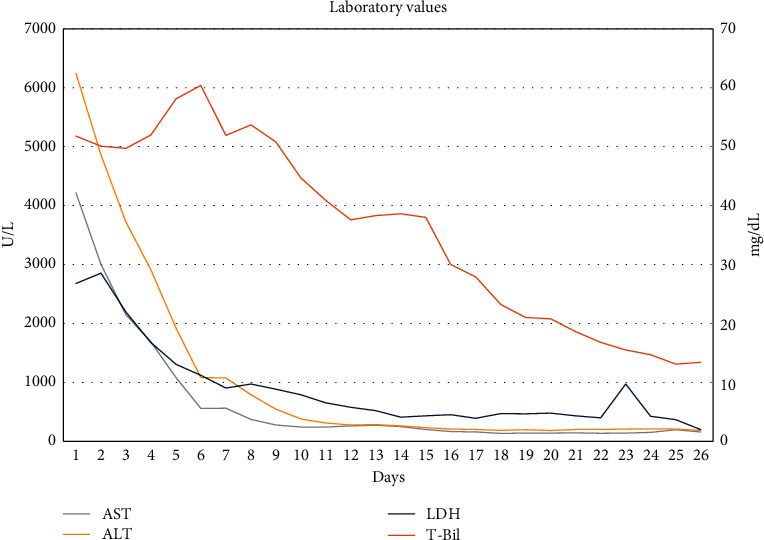
Changes in AST, ALT, LDH, and T-Bil during hospitalization are plotted from admission at days 1–25.

**Figure 4:**
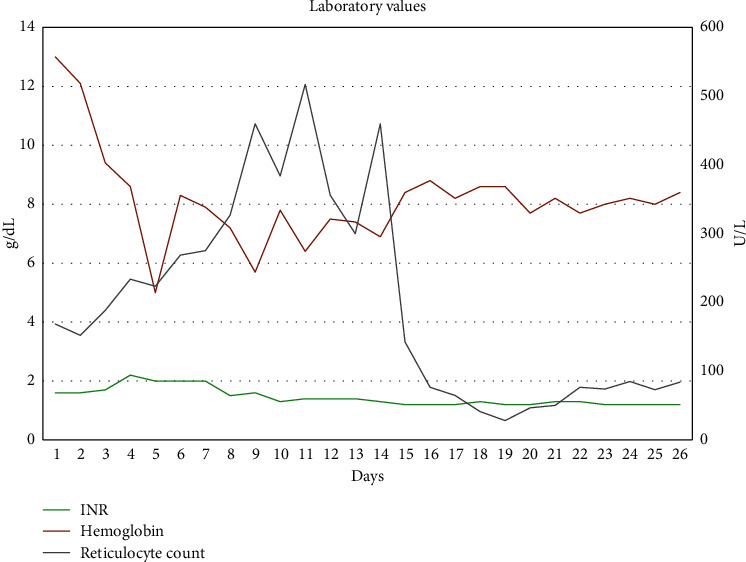
Changes in INR, hemoglobin, and reticulocyte counts during hospitalization are plotted from admission at days 1–25.

The patient was treated empirically with antibiotics and intravenous fluids. Patient continued to be oliguric and hemodialysis was initiated. Peripheral smear showed fragmented erythrocytes, spherocytes, bite cells, and target cells indicative of hemolysis. Fibrinogen was normal, and Coomb's test was negative. Liver biopsy showed cholestatic hepatitis with areas of resolving necrosis (Figure 1). Renal biopsy showed acute tubular injury with bilirubin and hemoglobin casts (Figure 2). The patient was initially listed for transplant but clinically improved with supportive measures alone. Over the next few weeks, patient's liver functions test and hemolysis labs steadily improved (Figures 3 and 4). He was discharged to a rehab facility and has since followed up in our clinic. His most recent liver function tests, 3 months after hospitalization, are normal. Erythrocyte G6PD is 3.3 U/g Hb, confirming the diagnosis of G6PD deficiency. His renal function also improved, and he is no longer on dialysis.

## 3. Discussion

Hepatitis A is the most common cause of viral hepatitis worldwide, generally causing a mild self-limited illness akin to other foodborne illnesses. Chronic sequelae are rare, and mortality related to hepatitis A accounts for only 0.5% of deaths secondary to viral hepatitis [10]. However, recent data suggest a dramatic increase in the number of outbreaks occurring worldwide. The recent CDC morbidity and mortality report warn of a considerable increase in hepatitis A in the United States, with cases climbing by 294% in 2016–2018 compared to the country's nadir in 2013–2015 [12, 13]. Acute liver failure secondary to hepatitis A is rare, but with higher rates in older adults >40 years of age or those with chronic liver disease [10, 14]. Hemolytic anemia has been associated with viral hepatitis, but the degree is usually mild to moderate [3]. When the disease course is complicated by severe hemolysis, a diagnosis in addition to hepatitis should be sought out [3, 15]. A more common occurrence seemed to be the finding of underlying G6PD deficiency in patients who developed severe hemolysis [2–4, 6–9, 15].

To our knowledge, this is the first reported case of severe hemolysis and renal failure precipitated by acute HAV in an undiagnosed G6PD deficient and HIV-positive patient in the United States. G6PD deficiency is the most common human enzyme defect, affecting more than 400 million people worldwide. This X-linked hereditary defect impairs catalyzation of the first step in the pentose phosphate pathway, which is crucial for the production of NADPH, protecting cells from oxidative stress, and regenerating glutathione for reduction of reactive oxygen species [16, 17]. While usually a clinically silent disorder, certain stressors, including drugs (particularly primaquine or trimethoprim-sulfamethoxazole), ingestion of food (fava beans, bitter melon, blueberries, and falafel), or viral infection can trigger an acute hemolytic crisis [18–20]. The global prevalence of the disorder is 4.9%, with African and Mediterranean descent at the highest risk [21], an estimated 10% of black Americans and as high as 70% of Kurdish Jews may carry the mutation [16].

Despite a high prevalence of G6PD deficiency in the African American population, there are no current universal screening guidelines for the disease. However, evidence suggests that these patients have a more complicated course when infected by hepatitis A than a non-G6PD deficient patient [3, 5, 22]. In a retrospective study by Gotsman and Muskat of patients with acute hepatitis A admitted between 1980 and 1999, those with G6PD deficiency demonstrated a more severe clinical course with severe hemolysis and higher bilirubin level [5]. It has been hypothesized that G6PD deficient hepatocytes have reduced levels of glutathione, thereby causing an accumulation of free radicals and delayed hepatocyte repair with viral insult. There is ongoing discussion regarding the utility of blood transfusions or plasmapheresis in cases with severe hemolysis to prevent long-standing complications including renal failure [8].

On review of literature, there are 80 cases related to viral hepatitis, and G6PD deficiency causing severe hemolysis has been reported (Table 1) [2–9, 23–35]. The patient's median age was 19.5 years (IQR: 14.25–35). Among these patients, 32 had hepatitis A, 19 had hepatitis E, and 29 had unspecified viral hepatitis as a precipitating cause for severe hemolysis. 22.5% (18/80) of the patients had renal failure as a complication, and of that, 12 were treated with dialysis. One patient was treated with plasmapheresis. Of the 80 total patients, 72 (90%) made a full recovery, while 8 died of complications from renal/liver failure, seizures, sepsis, and cerebral hemorrhage. Our case was an immunocompromised patient with HIV treated with empiric antibiotics, blood transfusion, and hemodialysis with subsequent clinical improvement.

With a large population at risk and an alarming rise in acute HAV cases in the United States, it is of utmost importance for clinicians to be cognizant of typical and atypical features of the infection. Although most acute HAV cases will resolve with supportive care alone, those with underlying G6PD deficiency are at an increased risk for developing severe intravascular hemolysis, marked hyperbilirubinemia, and increased risk of developing acute renal failure. Oxidant medications and nephrotoxins should be avoided. Treatment is generally supportive, but corticosteroids and plasma exchange can be considered in certain instances [15]. We recommend the hepatitis A vaccine in all patients with underlying G6PD deficiency. Ultimately, a high clinical suspicion and early recognition of patients at risk for severe manifestations of viral hepatitis can alter the course of the disease and aid in preventing chronic sequelae.

## 4. Conclusions

While this may be the first reported case with an HIV-positive patient in the United States, the phenomenon is one that has been reported in foreign literature for decades. With a high prevalence of G6PD deficiency and an increased incidence of hepatitis A in the past few years, it is imperative for clinicians to be mindful of the potential complications to prevent chronic sequelae and adverse outcomes. In conclusion, intravascular hemolysis due to G6PD deficiency should be considered in patients with acute hepatitis A infection who present with marked hyperbilirubinemia and renal failure.

## Figures and Tables

**Table 1:** Summary of reported cases with concomitant viral hepatitis and G6PD deficiency causing severe hemolysis.

Year	Author	Number of cases	Age (y)	Sex	Presenting symptoms	Geographic region	Cause of viral hepatitis	Presence of renal failure	Treatment	Need for transplant	Outcome
1966	Salen [23]	3	38, 28, 39	F, M, M	Fever, anorexia, and dark urine	USA	Did not specify	Yes (1 patient)	F: peritoneal dialysis, prednisone 40 mg qday; M-28: IVF; M-39: IVF and blood transfusion	No	Full recovery
1969	Clearfield et al. [4]	3	29, 31, 24	M, M, M	Myalgia, nausea, pruritis, dark urine, and jaundice	USA	Did not specify	No	Supportive care for all three patients	No	Full recovery
1975	Chan and Todd [3]	18	N/A	N/A	Fever and jaundice	Hong Kong	Did not specify	No	Supportive care for all 18 patients	No	15 had full recovery and 3 died from renal failure and hemolysis
1985	Agarwal et al. [24]	5	9, 19, 10, 10, 8	5 M	Fever, jaundice, and vomiting	India	Did not specify	Yes (2 patients)	4 patients required blood transfusions and 1 patient required peritoneal dialysis, blood transfusions, and antibiotics	No	4 had full recovery and 1 died of liver and renal failure
2001	Gotsman Muszkat [5]	18	Average 15.1	13 M, 5 F	Fever and jaundice	Israel	A	No	3 patients required blood transfusion, IVF fluids, and antibiotics. 15 patients required supportive care	No	Full recovery
2002	Abid and Khan [25]	5	26,16, 14, 22, 35	5 M	Fatigue, fever, jaundice, and vomiting	Pakistan	E	Yes (5 patients)	Supportive care for all 5 patients and 2 patients required hemodialysis	No	Full recovery
2003	Monga et al. [26]	1	35	M	Fever, abdominal pain, fatigue, jaundice, and dark urine	India	E	No	Supportive care	No	Full recovery
2008	Ozbay Hosnut et al. [8]	2	10, 5	M, F	Jaundice, emesis, and abdominal pain	Europe	A	Yes (1 patient)	Male: IVF, blood transfusion, and plasmapheresis; female: antibiotic therapy, vitamin K, lactulose, IVF, and blood transfusion	No	Full recovery
2009	Thapa et al. [27]	1	7	M	Jaundice	India	E	No	Supportive care	No	Full recovery
2011	Somani et al. [29]	1	17	M	Fever, abdominal pain, anorexia, jaundice, and dark urine	India	E	Yes	Supportive care	No	Full recovery
2011	Au et al. [28]	1	54	M	Jaundice, cyanosis, and confusion	Hong Kong	E	Yes	Hemodialysis	No	Died of cerebral hemorrhage
2012	Jain et al [6]	10	Average 16.1	8 M, 2 F	N/A	India	A, E	No	Supportive care	No	Full recovery
2012	Wing-Yan and Chan [34]	2	53, 54	M	Right upper quadrant pain and flu-like symptoms	Hong Kong	E	Yes (1 patient)	Blood transfusion and hemodialysis	No	53-year-old died of seizures and sepsis. 54-year-old died of renal and liver failure
2014	Tomar et al. [31]	1	15	M	Fever, jaundice, dark urine, nausea, fatigue, and decreased PO intake	India	E	Yes	Blood transfusion and hemodialysis	No	Full recovery
2015	Dea [30]	1	20	M	Fever, jaundice, and dark urine	India	A	No	Supportive care	No	Full recovery
2016	Karki et al. [32]	1	48	M	Jaundice, dark urine, fever, and confusion	India	E	Yes	Blood transfusion, hemodialysis, and antibiotics	No	Full recovery
2017	Moiz and Ali [7]	1	10	F	Fever, abdominal pain, and lethargy	Pakistan	A	No	Supportive care	No	Full recovery
2017	Ahmad et al. [33]	1	28	M	Jaundice and abdominal pain	Pakistan	E	Yes	Blood transfusion and hemodialysis	No	Full recovery
2018	Sharma et al. [9]	1	39	M	Fever, jaundice, and dark urine	India	A	Yes	Blood transfusion and intermittent hemodialysis	No	Full recovery
2019	Bajpai et al. [2]	1	16	M	Fever, jaundice, and dark urine	India	A	Yes	IVF, ventilation, antibiotics, continuous renal replacement, and plasma and blood transfusion	No	Full recovery
2020	Kamani et al. [35]	2	19, 15	2 M	Jaundice, nausea, and vomiting	Pakistan	E	No	IVF and blood transfusion	No	19-year-old died from liver failure. 15-year-old made a full recovery
2020	Abutineh et al. (present case)	1	55	M	Fatigue, fever, malaise, jaundice, and abdominal pain	US	A	Yes	IVF, antibiotics, blood transfusion, and hemodialysis	No	Full recovery

## Abbreviations

ART: Antiretroviral therapy
ATN: Acute tubular necrosis
G6PD: Glucose-6-phosphate dehydrogenase
HBV: Hepatitis B virus
HIV: Human immunodeficiency virus.

## References

[1] Havelaar A. H. (2015). World Health Organization Global estimates and regional comparisons of the burden of foodborne disease in 2010. *PLoS Medicine*.

[2] Bajpai M., Kakkar B., Patale D. (2019). Role of high-volume plasma exchange in a case of a G6PD deficient patient presenting with HAV related acute liver failure and concomitant acute renal failure. *Transfusion and Apheresis Science*.

[3] Chan T. K., Todd D. (1975). Haemolysis complicating viral hepatitis in patients with glucose-6-phosphate dehydrogenase deficiency. *Bmj*.

[4] Clearfield H. R., Brody J. I., Tumen H. J. (1969). Acute viral hepatitis, glucose-6-phosphate dehydrogenase deficiency, and hemolytic anemia. *Archives of Internal Medicine*.

[5] Gotsman I., Muszkat M. (2001). Glucose-6-phosphate dehydrogenase deficiency is associated with increased initial clinical severity of acute viral hepatitis A. *Journal of Gastroenterology and Hepatology*.

[6] Jain A. K., Sircar S., Jain M., Adkar S., Waghmare C., Chahwala F. (2013). Increased morbidity in acute viral hepatitis with glucose-6-phosphate dehydrogenase deficiency. *Indian Journal of Gastroenterology*.

[7] Moiz B., Ali S. A. (2018). Fulminant hemolysis in glucose-6-phosphate dehydrogenase deficiency. *Clinical Case Reports*.

[8] Ozbay Hosnut F., Ozcay F., Selda Bayrakci U., Avci Z., Özbek N. (2008). Etiology of hemolysis in two patients with hepatitis A infection: glucose-6-phosphate dehydrogenase deficiency or autoimmune hemolytic anemia. *European Journal of Pediatrics*.

[9] Sharma D., Singh O, Juneja D, Goel A, Garg S. K, Shekhar S (2018). Hepatitis A virus-induced severe hemolysis complicated by severe glucose-6-phosphate dehydrogenase deficiency. *Indian Journal of Critical Care Medicine : Peer-Reviewed, Official Publication of Indian Society of Critical Care Medicine*.

[10] Shin E. C., Jeong S. H. (2018). Natural history, clinical manifestations, and pathogenesis of hepatitis A. *Cold Spring Harbor Perspectives in Medicine*.

[11] Serious Hepatitis A. (1998). An analysis of patients hospitalized during an urban epidemic in the United States. *Annals of Internal Medicine*.

[12] Nelson N. P. (2020). Prevention of hepatitis A virus infection in the United States. *Recommendations of the Advisory Committee on Immunization Practices*.

[13] Foster M. A., Hofmeister M. G., Kupronis B. A. (2019). Increase in Hepatitis a virus infections-United States, 2013–2018. *MMWR. Morbidity and Mortality Weekly Report*.

[14] Lemon S. M. (2017). Type a viral hepatitis: a summary and update on the molecular virology, epidemiology, pathogenesis and prevention. *Journal of Hepatology*.

[15] (1966). Acute hemolytic anemia complicating viral hepatitis in patients with glucose-6-phosphate dehydrogenase deficiency. *Annals of Internal Medicine*.

[16] Beutler E. (1994). G6PD deficiency. *Blood*.

[17] Cappellini M., Fiorelli G. (2008). Glucose-6-phosphate dehydrogenase deficiency. *The Lancet*.

[18] Luzzatto L., Arese P. (2018). Favism and glucose-6-phosphate dehydrogenase deficiency. *New England Journal of Medicine*.

[19] Babu T., Panachiyil G. M., Sebastian J., Ravi M. D. (2019). Probable blueberry-induced haemolysis in a G6PD deficient child: a case report. *Nutrition and Health*.

[20] Stone S. N., Reisig K. V., Saffel H. L., Miles C. M. (2020). Management of athletes with G6PD deficiency: does missing an enzyme mean missing more games?. *Sports Health: A Multidisciplinary Approach*.

[21] Nkhoma E. T., Poole C., Vannappagari V., Hall S. A., Beutler E. (2009). The global prevalence of glucose-6-phosphate dehydrogenase deficiency: a systematic review and meta-analysis. *Blood Cells, Molecules, and Diseases*.

[22] Kattamis C. A., Tjortjatou F. (1970). The hemolytic process of viral hepatitis inchildren with normal or deficient glucose-6-phosphate dehydrogenase activity. *The Journal of Pediatrics*.

[23] Salen G. (1966). Acute hemolytic anemia complicating viral hepatitis in patients with glucose-6-phosphate dehydrogenase deficiency. *Annals of Internal Medicine*.

[24] Agarwal R. K., Moudgil A., Kishore K., Srivastava R. N., Tandon R. K. (1985). Acute viral hepatitis, intravascular haemolysis, severe hyperbilirubinaemia and renal failure in glucose-6-phosphate dehydrogenase deficient patients. *Postgraduate Medical Journal*.

[25] Abid S., Khan A. H. (2002). Severe hemolysis and renal failure in glucose-6-phosphate dehydrogenase deficient patients with hepatitis E. *The American Journal of Gastroenterology*.

[26] Monga A., Makkar R. P., Arora A., Mukhopadhyay S., Gupta A. K. (2003). Case report: acute hepatitis E infection with co-existent glucose-6-phosphate dehydrogenase deficiency. *Canadian Journal of Infectious Diseases*.

[27] Thapa R., Pramanik S., Biswas B., Mallick D. (2009). Hepatitis E virus infection in a 7-year-old boy with glucose 6-phosphate dehydrogenase deficiency. *Journal of Pediatric Hematology/oncology*.

[28] Au W. Y., Ngai C.-W., Chan W.-M., Leung R. Y. Y., Chan S.-C. (2011). Hemolysis and methemoglobinemia due to hepatitis E virus infection in patient with G6PD deficiency. *Annals of Hematology*.

[29] Somani S. K. (2011). Hepatitis e virus infection leads to severe hemolysis in glucose-6-phosphate dehydrogenase deficiency patients.

[30] Dea B. (2015). Severe hemolysis as the first manifestation of acute hepatitis A in an adult with G6PD deficiency and positive ANA. *American Journal of Medical Case Reports*.

[31] Tomar L. R., Aggarwal A., Jain P., Rajpal S., Agarwal M. P. (2015). Acute viral hepatitis E presenting with haemolytic anaemia and acute renal failure in a patient with glucose-6-phosphate dehydrogenase deficiency. *Tropical Doctor*.

[32] Karki P., Malik S, Mallick B, Sharma V, Rana S. S (2016). Massive Hemolysis causing renal failure in acute hepatitis E infection. *Journal of Clinical and Translational Hepatology*.

[33] Ahmad B. S., Ahmad A, Jamil S, Abubakar Mohsin Ehsanullah S. A, Munir A (2018). Severe haemolysis and renal failure precipitated by hepatitis E virus in G6PD Deficient patient: a case report. *JPMA. The Journal of the Pakistan Medical Association*.

[34] Wing-Yan A., Chan S. (2012). Association between glucose 6-phosphate dehydrogenase (G6PD) deficiency and fatal outcome. *Singapore Medical Journal*.

[35] Kamani L., Shaikh H., Khemchandani A. K. (2020). Fulminant hepatic failure in glucose-6-phosphate dehydrogenase (G6PD) deficient patients caused by hepatitis E infection: a single disease with different spectrums. *Cureus*.

